# H1299R Variant in Factor V and Recurrent Pregnancy Loss: A Systematic Review and Meta-Analysis Protocol

**DOI:** 10.3390/genes13061019

**Published:** 2022-06-06

**Authors:** Alessio Ardizzone, Anna Paola Capra, Stefania Mondello, Silvana Briuglia, Maria Angela La Rosa, Michela Campolo, Emanuela Esposito

**Affiliations:** 1 Department of Chemical, Biological, Pharmaceutical, and Environmental Sciences, University of Messina, Viale Ferdinando Stagno D’Alcontres 31, 98166 Messina, Italy; aleardizzone@unime.it (A.A.); annapaola.capra@unime.it (A.P.C.); campolom@unime.it (M.C.); 2 Department of Biomedical and Dental Sciences and Morphofunctional Imaging, University of Messina, Via Consolare Valeria 1, 98125 Messina, Italy; stefania.mondello@unime.it (S.M.); silvana.briuglia@unime.it (S.B.); 3 Genetics and Pharmacogenetics Unit, “Gaetano Martino” University Hospital, Via Consolare Valeria 1, 98125 Messina, Italy; maria.larosa@unime.it

**Keywords:** thrombophilia, thrombophilic gene variants, factor V, H1299R, A4070G, recurrent pregnancy loss (RPL), miscarriages

## Abstract

Recurrent pregnancy loss (RPL) is defined as the loss of two or more pregnancies, affecting approximately 1 to 3% of women worldwide. Scientific data highlight a possible correlation between thrombophilic genetic variants and RPL. H1299R variant in the factor V gene would lead to an increased thrombotic risk associated with frequent miscarriages. However, the data are often conflicting, making this an interesting question for further investigations by evaluating genotype-phenotype correlations to improve the clinical management and genetic counseling of couples. A systematic review and meta-analysis will follow the preferred reporting elements for systematic review and meta-analysis protocols (PRISMA-P). The Pubmed (MEDLINE) and Embase (OVID) databases will be explored to identify suitable articles based on inclusion and exclusion criteria. Inclusion criteria are: (a) H1299R genotyping with clear data reported, referred to as Heterozygous (Het) and/or Homozygous (Hom); (b) articles written in English; (c) analyses of only RPL female patients having at least two or more previous pregnancy losses and compared with a control group. This analysis will present selected scientific evidence, addressing the questions concerning the association between the H1299R variant and RPL, hoping to clarify this still unresolved issue. PROSPERO registration number: CRD42022330077.

## 1. Introduction

Human reproduction is a process extremely delicate, in which a vast complexity of biological, hereditary, and environmental factors plays an important part [1,2]. Although some processes underlying fertility and non-successful pregnancies have been understood, the etiology of approximately half of RPL cases remains not identified [3].

It is known how the risk of spontaneous abortion increases with advancing maternal age, but many factors remain largely unidentified.

Pregnancy loss can be considered a quite common complex trait with a genetic risk factor involved [4,5].

Most of the studies have focused on the associations of maternal genetic variants with recurrent spontaneous abortion, often reporting inconclusive results and failing to estimate the possible risk of recurrence after a first abortion [6].

The up-to-date guidelines of ESHRE, the European Society of Human Reproduction and Embryology, report 38 suggestions on risk factors, prevention, and clinical management in couples with recurrent pregnancy loss (RPL) [7]. ESHRE guidelines elucidate possible predisposing genetic conditions would already come into play and would be worthy of careful investigation.

In particular, clinicians should pay attention to the medical conditions present in the family and the reproductive history of the couple. Based on the ESHRE recommendations, both the number of previous abortions and the woman’s age represent important information for the prognosis of RPL patients [7].

Similarly, the American Society for Reproductive Medicine (ASRM) and Royal College of Obstetricians and Gynecologists (RCOG) reported guidelines using different definitions for RPL. It recommended criteria to investigate the genetic, anatomical, hormonal, infectious, and thrombophilic factors involved [8].

Thrombophilia is an abnormal blood coagulation condition leading to a hypercoagulability status. Thrombophilia can be an inherited or acquired tendency to form blood clots, commonly manifested as an increased risk of venous thromboembolism (VTE) [9,10].

Pregnancy difficulties such as RPL and recurrent implantation failure have been related to thrombophilic gene variants [11,12].

Particularly, scientific findings advised an increased risk of RPL in pregnant women with hereditary thrombophilia compared with the corresponding control group.

However, the link between hereditary thrombophilia conditions and RPL is still discordant due to the divergences in sample size, nationality, and other aspects investigated by the insufficient studies performed.

Of interest, reported variants in the coagulation *factor V* (*FV*) gene, a single chain procofactor that acts in regulating blood coagulation with other plasma factors, are V Leiden variant, c.1691G>A substitution resulting in Arg in Glu replacement at amino acid 506 (R506Q) (rs6025); *FV* A4070G (R2 allele: H1299R) (rs1800595); FV Hong Kong and Cambridge, associated with substitution of Arg306 by Gly (FV Hong Kong) and Thr (FV Cambridge) [13,14,15,16]. However, FV Cambridge or FV Hong Kong variants are not detected in either control or RPL patient groups, respectively, from Asians and Africans [15,17].

In contrast, the well-known mutation Factor V Leiden causes Activated Protein C (APC) resistance determining a hypercoagulable state, which constitutes a lifelong risk factor for thrombosis and pregnancy complications, such as RPL [13,14]. Another missense variation in exon 13 of the *FV* gene, known as A4070G; His1299Arg, R2, was identified and linked to hereditary thrombophilia. Previous studies have shown that His1299Arg change will increase 2–3 folds the VTE risk [13].

Therefore, based on this evidence, the objective of this study protocol will be to lay the foundation for an updated and exhaustive systematic review and meta-analysis on the relation between hereditary thrombophilia variant H1299R and its association with the risk of RPL.

## 2. Materials and Methods

**Figure 1:**
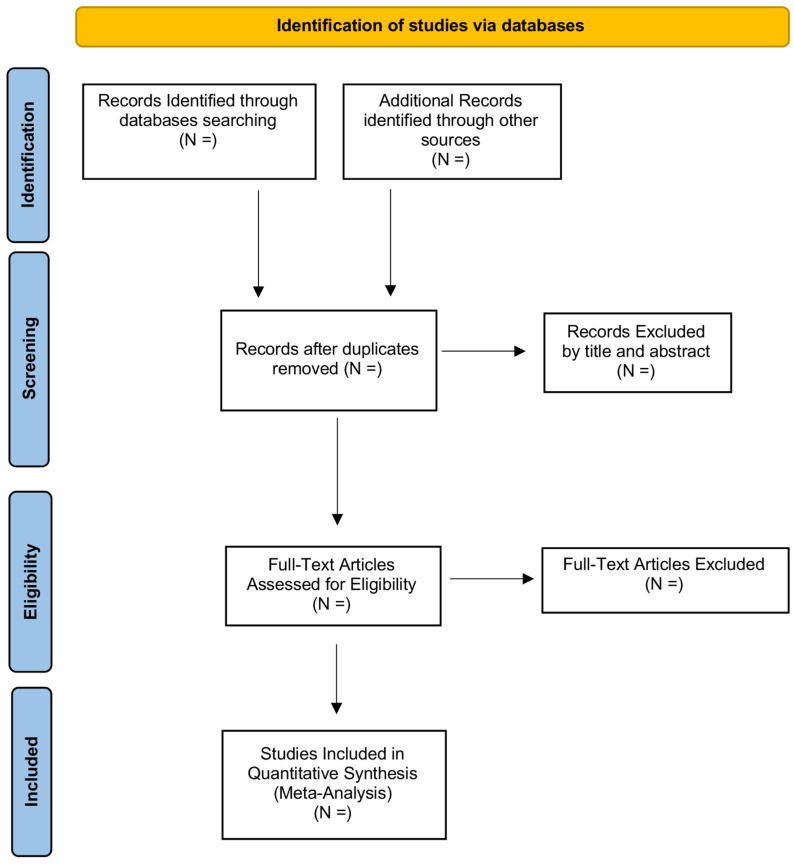
Preferred Reporting Items for Systematic Review and Meta-analysis (PRISMA) flowchart of searching and screening.

This review protocol has been registered in the PROSPERO international prospective register of systematic reviews; the registration number is CRD42022330077. We will use The Preferred Reporting Items for Systematic Review and Meta-analysis Protocols (PRISMA-P) as guidelines for reporting present protocol and consequent meta-analysis. Consequently, we will illustrate the screening process through the (PRISMA-P) flowchart, thus reporting the preferred items for systematic review and meta-analysis (see Figure 1).

We will serve online databases as the principal method to identify eligible studies. Moreover, we will also implement a manual search of relevant papers. The study will also conduct reference research back to other reviews and consult experts in medical genetics and biostatistics.

### 2.1. Data Sources and Search Strategy

The bibliographic databases MEDLINE (Pubmed) and EMBASE (Ovid) will be searched for literature clinical studies that evaluated the correlation between the presence of variant H1299R in Heterozygosity (Het) or Homozygosity (Hom) and RPL. Both databases will be investigated for terms related to thrombophilia genes and RPL. Citations, titles, and abstracts will be exported into Endnote X9. The search plan will be aligned with the support of the Cochrane handbook. Keywords combinations used during the search strategy are scheduled in Table 1.

Original clinical articles that report a numerical and well-distinguished (Het or Hom) presence of the H1299R variant will be included. We will consider observational studies, case-control studies, cross-sectional studies, and cohorts.

Specifically, during the selection of studies, we will also consider the following inclusion criteria:

- Papers analyzing only females with RPL, studies on couples will not be considered.
- RPL patients having at least two or more previous pregnancy losses compared to a healthy control group with at least one successful pregnancy; studies using male patients or literature data as control will not be eligible.
- Only articles written in English; articles that aren’t available in English will be excluded.

No geographic exclusion criteria or temporary restrictions will be imposed. The searches will be re-run just before the final analyses and further studies retrieved for inclusion.

Editorials, letters, reviews, guidelines, case reports, abstracts and conference proceedings, systematic reviews and meta-analyses, and ongoing studies will be excluded.

Studies will also be excluded if they did not report the year in which the measure of occurrence was estimated, the data are not clearly reported (e.g., the distinction not clear between Het and Hom, e.g., unclear number of patients analyzed, e.g., no comparison with a healthy control group).

Data on the following items will be collected: data source, study population, study years, study design, H1299R outcomes description as number of patients with H1299R variant or wild type, geographical area, mean age of patients, number of previous abortions for the RPL group. Our future systematic review and meta-analysis will be based on the outcomes of previous clinical studies. Subsequently, ethical approval is not necessary. Further, the results will be submitted to a peer-reviewed journal of the relative field for consideration for publication.

### 2.2. Study Selection, Data Extraction, and Assessment of Risk of Bias

Afterward, duplicates will be removed from the two different databases; two review authors will screen individually the titles and abstracts of all records identified to discard articles that will be off-topic. After this, full-text articles will be scrutinized to determine their eligibility for inclusion in the systematic review and meta-analysis. Every divergence will be resolved through the intermediation of a third review author.

The Newcastle Ottawa Scale (NOS) recommended by the Agency for Healthcare Research and Quality will be employed to assess the quality of articles, as previously reported by Ingrosso et al. [18]. A total of 9 key criteria will be judged. The total score will be 9. The following ranges will be used as standard: score < 4 “high risk of bias”, a score between 4–6 “intermediate risk of bias”, and a score between 6–9 “low risk of bias”.

### 2.3. Data Analysis Procedure

As far as we know, there are enough studies in the literature, so the likelihood of a meta-analysis is well supported. The meta-analysis will include four procedures: first, the evaluation of the risk of bias for each study; second, data acquisition and consequent correlation between the H1299R variant and RPL; third, to compare, through separate assessments (subgroups analysis), the correlation between H1299R and RPL based on geographic origin and the mean age as parameters; finally, sensitivity analysis will be performed. Review Manager (RevMan. Version 5.4. Copenhagen: The Nordic Cochrane Centre, The Cochrane Collaboration, 2014) will be used to perform a meta-analysis of the extracted data in the systematic review.

Odds ratio (OR) will be employed as a statistical measure of the association between the H1299R variant and RPL. Random-effects model meta-analysis will be applied to calculate the pooled ORs and 95% CIs [19].

To estimate the heterogeneity between the studies, the Chi^2^ and the I^2^ statistics will be evaluated. *p* < 0.10 denote statistically significant heterogeneity, and I^2^ statistic will be evaluated based on the following criteria: I^2^ > 75% indicates an extremely high degree of heterogeneity, I^2^ 51–75% indicates a high degree of heterogeneity, I^2^ 26–50% indicates a moderate-degree of heterogeneity, and I^2^ ≤ 25% indicates a low degree of heterogeneity [20,21].

The recommendation of Cushing et al. will be applied [22]. Based on this, a fixed-effects model will be used in the analysis if *p* > 0.01, I^2^ < 50%. Otherwise, a random-effects model will be applied in the case of high heterogeneity (*p* < 0.10, I^2^ > 50%) [22]. Furthermore, subgroup and sensitivity analyses will be employed to further investigate the influence of H1299R based on several factors or removing high-risk bias studies, respectively.

### 2.4. Subgroup and Sensitivity Analyses

The purpose of the subgroup analysis will be to validate the impact of the FV H1299R variant, considering additional parameters that could provide a different interpretation. Subgroup analysis will be conducted using the following characteristics: the geographic origin of the population and the average age of RPL patients.

One of the fundamental problems for the meta-analysis is the unavailability of some estimations from published and unpublished studies.

Disclosure bias could contribute to this incompleteness. For this reason, the possibility of carrying out sensitivity analysis should always be considered to allow for the most reliable conclusions.

In accordance, at the end of our study, we will perform a sensitivity analysis excluding the possible studies with “high-risk bias” or having obtained the lowest scores on the NOS scale.

This will allow us to estimate the overall effect more precisely and interpret the results obtained more appropriately.

## 3. Discussion

Spontaneous abortion indicates a sudden termination of pregnancy which usually occurs within 20–24 weeks of gestation [23].

Otherwise, ectopic pregnancies and molar pregnancies are not included in the definition of recurrent pregnancy loss [7,24].

Unfortunately, the percentage of cases in which a pregnancy loss occurs is greater than 20% of clinically recognized pregnancies [25], and in particular, most cases occur during the first trimester of gestation [26].

RPL, unlike sporadic abortion, is considered a disorder that requires medical intervention to ensure access to specialized clinics and thorough medical investigations, as well as increased assistance and monitoring in future pregnancies [24].

This event has an important emotional impact on the woman who experiences it, and in general, on the couple, who very often need psychological support to overcome the traumatic event [27,28].

The pathophysiology of RPL is based on several factors, including maternal age and gestational age, although various mechanisms can converge on a common pathway that triggers miscarriage [29,30].

Others risk factors that predispose to spontaneous abortion are viral infections, structural abnormalities of the reproductive organs, use of drugs or smoking, diabetes, and especially genetic anomalies [31,32,33,34,35].

Notably, common mechanisms of RPL include chromosomal errors in the conceptus that preclude further development and rupture of the maternal-fetal interface, both of which result in bleeding, cramping, and miscarriage. However, reliable risk factors have not yet been identified in over 50% of RPL women [36,37].

RPL represents a complex clinical picture in reproductive medicine, thus resulting in a clinical challenge for the affected women, their families, and the healthcare team [38]. Physicians and healthcare professionals should appropriately advise RPL patients, recommending thorough investigations and treatments [38]. Hence, interprofessional cooperation between endocrinologists, obstetricians, nurses, and geneticists plays a fundamental task in assisting couples.

In this perspective, careful and accurate genetic counseling could be an important step in evaluating couples planning a pregnancy to consider the presence of one or more individuals/familiars’ risk factors [39,40].

Numerous investigations analyzed the role of inherited thrombophilia variants by studying gene polymorphism associated with RPL [41].

It has been well investigated how chromosomal abnormalities induce implantation defects. In particular, the aberration in chromosomal structure can originate from deficiencies in the checkpoint mechanisms, which represent a quality control pool of the cell able to distinguish abnormalities of meiosis. This complex machine is called Assembly Checkpoint (SAC) and is formed by various proteins such as Mad1, Mad2, Bub1, Bub3, BubR1, and Mps1 [5]. Accordingly, another report elucidated the question of hypofertility and successful pregnancies by screening two candidate genes, aurora kinase B (AURKB) and synaptonemic complex protein 3 (SYCP3) [42]. About the *AURKB* gene, Lopez-Carrasco et al. discovered non-synonymous low-frequency (0.5%) variants (c. 155C>T, c. 236T>C, and c. 880G>A) that induce changes p.A52V, p. I79T and p.A294T in the polypeptide chain respectively; 236T>C and 880G>A linked with RPL [42].

These results are promising and underscore the need for additional efforts to investigate the association between RPL and genetic polymorphisms.

Our study will focus on the H1299R variant in FV, which significantly increases the risk of fetal loss during pregnancy [43]. Although these data are certainly worthy of further considerations, conflicting data seem to disprove this hypothesis [44].

Relatedly, recent studies have not observed any significant correlation between the factor V H1299R mutation and the incidence of RPL, thus confirming the high variability of results regarding the association of this polymorphism with RPL [45].

Based on this dualism, we believe that this study may lead to several clarifications for geneticists, gynecologists, obstetricians, and researchers.

Indeed, despite the discordant findings, clinicians continue to screen women aiming to clarify the combination between thrombophilic genetic variants and reverse pregnancy outcomes [44].

Thus, elucidating the associations between the H1299R variant and RPL through the combination of multiple clinical studies could represent an important goal in pregnancy and childbirth research.

Furthermore, our study will aim to evaluate the impact of the H1299R heterozygous and homozygous variants carefully and separately, in order to carry out a comprehensive analysis of the genetic risk associated with RPL.

In addition, the subgroup analysis will allow us to estimate the influence of the H1299R variant on RPL in relation to the geographic area (e.g., Africa, Asia, Europe, America, and Oceania) and the mean age of analyzed RPL patients.

In addition, a sensitivity analysis will be performed by estimating the outcome, excluding high-risk bias studies.

## 4. Conclusions

The comprehension of thrombophilic genetic variants in RPLs represents a complex topic in which several parameters should be considered. To the best of our knowledge, this will be the first comprehensive systematic review and meta-analysis to shed light on the specific influence of the H1299R variant in the *FV* gene on RPL. Therefore, the present study will have a key role in providing evidence for future research in examining hereditary thrombophilia variant H1299R and recurrent miscarriage. This could also be a valid support for specified clinical centers and genetic counselors, thus remarking the importance of genetic screening and counseling for all couples who desire a child.

## Figures and Tables

**Table 1:** Search strategy.

Concept	Keywords
THROMBOPHILIA VARIANT “H1299R”	H1299R; His1299Arg; A4070G; 4070A/G; *FV* A4070G; *Factor V*; *FV*; *F5*; *Factor V* R2; FV R2; R2 FV; Factor V HR2; FV HR2; FV HR2 haplotype; Factor V HR2 (4070A/G); Factor V A1299H (A4070G); Factor V 4070 A/G; His1327Arg; 3980A>G.
PREGNANCY LOSS	Pregnancy Loss; Recurrent Pregnancy Loss; RPL; Miscarriage; Miscarriages; Abortion; Abortions; woman; women; Spontaneous abortion; Spontaneous abortions; recurrent fetal loss; recurrent fetal losses; fetal loss; fetal losses; intrauterine fetal death

## Data Availability

Not applicable.

## Abbreviations

RPL	Recurrent Pregnancy Loss
HET	Heterozygous
HOM	Homozygous
PRISMA-P	preferred reporting elements for systematic review and meta-analysis protocols
VTE	venous thromboembolism
FV	factor V
NOS	Newcastle Ottawa Scale
OR	Odds ratio
